# Supplementary material for: Enhancing patient-centered care: Evaluating quality of life in type 2 diabetes management
Source: PLoS One. 2025 Mar 11;20(3):e0319369. doi: 10.1371/journal.pone.0319369 (PMC11896040; doi:10.1371/journal.pone.0319369)
Supplement: S1 Table — (DOCX) [file pone.0319369.s001.docx]

| **Characteristic** | **Category** | **Frequency (%)** |
| --- | --- | --- |
| **Gender** | Male | 74 (49.0) |
|  | Female | 77 (51.0) |
| **Age** | Mean ± SD | 60.05 ± 11.63 |
|  | Range | 34–84 years |
| **Education Level** | No elementary education | 6 (4.0) |
|  | Primary education | 22 (14.6) |
|  | Secondary education | 69 (45.7) |
|  | Higher education | 19 (12.6) |
|  | University degree | 17 (11.3) |
| **Marital Status** | Married | 83 (54.7) |
|  | Widowed | 25 (16.7) |
|  | Living alone | 20 (13.3) |
|  | Divorced | 18 (12.0) |
| **Duration of Diabetes** | <5 years | 51 (34.0) |
|  | 5–10 years | 36 (24.0) |
|  | >10 years | 64 (42.0) |
| **Comorbidities** | None | 97 (64.0) |
|  | Present | 54 (36.0) |

**Table 1: Demographic Characteristics of Participants**
