# Supplementary material for: Enhancing patient-centered care: Evaluating quality of life in type 2 diabetes management
Source: PLoS One. 2025 Mar 11;20(3):e0319369. doi: 10.1371/journal.pone.0319369 (PMC11896040; doi:10.1371/journal.pone.0319369)
Supplement: S2 Table — (*Kruskal-Wallis test; **Mann-Whitney U test). (DOCX) [file pone.0319369.s002.docx]

|  | | Median | IQR |  | |
| --- | --- | --- | --- | --- | --- |
|  |  |  |  | **Z/H** | **p** |
| Gender | Male | 78,94 | 74,55-83,32 | 5,77** | 0,56 |
|  | Female | 80,32 | 75,17-85.14 |  |  |
| Duration of illnes | Less than 5 years | 84,79 | 80,93-88,65 | 4,62* | 0,09 |
|  | From 5 to 10 years | 76,04 | 66,99-95,09 |  |  |
|  | More than 10 years | 77,51 | 72,60-82,41 |  |  |
|  |  |  |  |  |  |
| Comorbidity | Yes | 75,20 | 70,34-80,05 | 2,84** | 0,00* |
|  | No | 85,52 | 82,37-88,67 |  |  |

**Table 2. Association between quality of life in T2DM patients and variables: gender, disease duration, and comorbidity. (*Kruskal-Wallis test; **Mann-Whitney U test).**
