# Supplementary material for: Enhancing patient-centered care: Evaluating quality of life in type 2 diabetes management
Source: PLoS One. 2025 Mar 11;20(3):e0319369. doi: 10.1371/journal.pone.0319369 (PMC11896040; doi:10.1371/journal.pone.0319369)
Supplement: S3 Table — (DOCX) [file pone.0319369.s003.docx]

|  | Age in years | Quality of life |
| --- | --- | --- |
| Pearson’s correlation(r) |  | -0.263 |
| Sig. (2-tailed) |  | 0,009 |
| N | 151 | 97 |

**Table 3. *The influence of age on the quality of life of respondents***
