# Supplementary material for: Enhancing patient-centered care: Evaluating quality of life in type 2 diabetes management
Source: PLoS One. 2025 Mar 11;20(3):e0319369. doi: 10.1371/journal.pone.0319369 (PMC11896040; doi:10.1371/journal.pone.0319369)
Supplement: S1 Questionnaire — (DOCX) [file pone.0319369.s004.docx]

Inclusivity in global research

PLOS’ policy on inclusivity in global research aims to improve transparency in the reporting of research performed outside of researchers’ own country or community and ensures that PLOS publications reporting global research adhere to high standards for research ethics and authorship. Authors of relevant research articles may be asked to complete the questionnaire below, which outlines ethical, cultural, and scientific considerations specific to inclusivity in global research. This questionnaire may be requested when researchers have travelled to a different country to conduct research, if research uses samples collected in another country, research with Indigenous populations or their lands, or if research is on cultural artefacts. Researchers travelling to another country solely to use laboratory equipment will not normally be required to complete the questionnaire. However, the questionnaire can be requested at the journal’s discretion for any submission – if you have been requested to complete this questionnaire by the PLOS journal you submitted to, please do so.

Please complete the questionnaire below and include this as a Supporting Information file with your manuscript. Note that if your paper is accepted for publication, this checklist will be published with your article in the supporting information files. Please ensure that you reference the checklist in the main body of your manuscript. We suggest adding a subsection ‘Inclusivity in global research’ to your Methods section and adding the following sentence: “Additional information regarding the ethical, cultural, and scientific considerations specific to inclusivity in global research is included in the Supporting Information (SX Checklist)”

The questions have been designed to be applicable to a wide range of study types, and there are subsections for both human subjects research and non-human subjects research. If any of the questions are not relevant to your research please mark them as “N/A” as appropriate.

**Ethical considerations, permits and authorship**

*This section is applicable to all research types.*

Provide details as to who granted permissions and/or consent for the study to take place in the Methods section of your manuscript. This should include the names of **all** ethics boards, governmental organizations, community leaders or other bodies that provided approval for the study. If individuals provided approval refer to these people by their role or title but do not list their name(s).

Reported on page number: 4

If there were any deviations from the study protocol after approval was obtained please provide details of these changes in the Methods section of your manuscript.
Did this study involve local collaborators that are residents of the country where the research was conducted or members of the community studied? If you do not have any authors from said communities, please provide an explanation for this below.

Reported on page number: No deviations from the study protocol after approval.

Thank you for your query regarding the involvement of local collaborators in our study titled "Enhancing patient-centered care: evaluating quality of life in type 2 Diabetes management."

We acknowledge the vital contributions of local collaborators in our research. Our team included doctors(co-authors) from the regions where the study was conducted. These collaborators were instrumental in designing the study, collecting data, and interpreting the results, ensuring that our approach was culturally sensitive and appropriately tailored to the specific needs of the community. Their insights and expertise have enriched the quality and applicability of our findings, making our study truly patient-centered.

Everyone listed as an author should meet PLOS’ criteria for authorship and all individuals who meet these criteria should be included in the author byline, rather than the acknowledgements. For further information please see the journal’s Authorship Policy.

**Human subjects research (e.g. health research, medical research, cross-cultural psychology)**

Did you obtain written informed consent from a representative of the local community or region before the research took place? How did you establish who speaks for the community? Details of written informed consent obtained from study participants should be reported separately in the Methods section of your manuscript.

Yes, written informed consent was taken, mentioned in page 4.

How did members of the local community provide input on the aims of the research investigation, its methodology, and its anticipated outcome(s)?

Our study, "Enhancing patient-centered care: evaluating quality of life in type 2 Diabetes management," actively involved local community members in several stages to ensure that our research was aligned with their needs and perspectives. Before finalizing the research aims and methodologies, we conducted a series of focus groups and community forums that included potential participants, local healthcare providers, and representatives from local diabetes support groups. These discussions helped us refine our research questions and tailor our methodology to better address the specific concerns and conditions of the community. For example, input received led to the inclusion of specific quality of life indicators that are particularly affected within the local context, which were not initially covered in our study design.

When engaging with the local community, how did you ensure that the informed consent documents and other materials could be understood by local stakeholders?

To ensure our informed consent documents were understandable and culturally appropriate, we implemented several key strategies:

1. **Translation and Back-Translation:** We had the consent forms translated into the local language by professional translators and then back-translated into English by a different team to ensure accuracy and preserve the original intent.
2. **Literacy Considerations:** We adapted our informed consent process to accommodate different literacy levels, offering both written and verbal explanations through trained bilingual staff who addressed any participant questions directly.
3. **Cultural Sensitivity:** Local cultural advisors helped us tailor the content and presentation of our materials to align with community norms and values, ensuring respectful and appropriate communication.
4. **Pilot Testing:** We conducted a pilot test of the informed consent process with community members, using their feedback to refine our approach before full deployment.

Will the findings of the research be made available in an understandable format to stakeholders in the community where the study was conducted (e.g. via a presentation, summary report, copies of publications, etc.)? Please provide details of how this will be achieved.

We are committed to ensuring that the outcomes of our study on "Enhancing patient-centered care: evaluating quality of life in type 2 Diabetes management" are accessible and understandable to the community stakeholders involved. The following steps outline how we plan to achieve this:
**Summary Reports:** Easy-to-read summary reports of the study findings will be prepared and distributed. These reports will include key insights, graphics, and recommendations in the local language to ensure comprehensibility.
**Feedback Sessions:** After the dissemination, we will hold feedback sessions to answer any questions and gather community responses to the findings. This will also help us gauge the impact of the information shared and further community engagement.

**Non-human subjects research using specimens/ animals collected as part of the study, or those housed in archival collections. Examples include archaeology, paleontology, botany and zoology.**

Did the permission you obtained from a local authority to perform the study include an agreement on access to outputs and benefit sharing? This may include procedures to enable fair distribution of the benefits and resources arising from the research performed. Please include any details of Prior Informed Consent and Benefit Sharing Agreements obtained. These may be required by field-specific regulations, for example the Convention on Biological Diversity (CBD) and the associated Nagoya Protocol.

Not Applicable

If the material used in your study was imported, please A) provide the year it was imported and B) indicate whether permits were obtained to import/export the materials used, C) provide details of any permits obtained. If this information is not available, please indicate this.

Not Applicable

If you used archival specimens, please state how the material used in your study was acquired by the institute it is held in and provide details of any permits obtained for the original excavations/ sample collection. If this information is not available, please indicate this.

Not Applicable

How was the potential cultural significance of the materials collected in your study to local communities considered in your research design? Were Indigenous peoples and/or local researchers and institutions involved with archaeological excavations / collection of specimens? If so, please provide a description of their involvement.

Not Applicable

If your manuscript includes photographs of human remains please indicate whether authors obtained permission from descendants or affiliated cultural communities to do so.

Not Applicable
