# Supplementary material for: Enhancing patient-centered care: Evaluating quality of life in type 2 diabetes management
Source: PLoS One. 2025 Mar 11;20(3):e0319369. doi: 10.1371/journal.pone.0319369 (PMC11896040; doi:10.1371/journal.pone.0319369)
Supplement: Attachment — Submitted filename: Reviewers comment.docx [file pone.0319369.s006.docx]

**Reply to the reviewers’ comments**

| Reviewer Number | Original comments of the reviewer | Reply by the author(s) | Changes done on page number and line number |
| --- | --- | --- | --- |
| Reviewer 1 | Introduction Clarity and Language Quality: The English is generally clear. However, some sentences could be more concise, and the flow could be improved by reducing redundancy and better organizing the information. Specific attention to transitions between topics would enhance readability. | as suggested: Introduction part is thoroughly changed. | Page 3  Line:94-101  Line:111-119 |
|  | Methods  Relevance: The Methods section is relevant, detailing the cross-sectional study design, the population studied, and the instruments used for data collection. However, the choice of the QLI and its adaptation for the Montenegrin population requires further justification, particularly regarding its specificity for diabetes-related QoL issues. | The choice of QLI and it’s adaptation for the Montenegrin population is justified and following sentence has been incorporated “We selected the Ferrans and Powers Quality of Life Index (QLI) for its ability to be customized for specific populations, making it highly relevant for assessing the impacts of Type 2 diabetes mellitus (T2DM) on quality of life. For our Montenegrin cohort, we adapted the QLI to focus on aspects crucial to diabetes management, such as dietary restrictions, medication adherence, and the psychological impacts of living with a chronic condition. This adaptation involved modifying the QLI based on consultations with local healthcare professionals and feedback from a pilot group of the study population, ensuring the tool's cultural and contextual relevance. These adjustments allow the QLI to accurately reflect the diabetes-related quality of life challenges specific to Montenegro, enhancing the instrument's sensitivity and the validity of our data.”  “ | Page 5: line 218-228 |
|  | Clarity and Language Quality: This section is straightforward but would benefit from more detailed descriptions of the data analysis procedures. The English is functional but could be enhanced by refining technical descriptions and using more precise statistical language to improve the professional tone. | More detail descriptions of data analysis has been incorporated. | Page 6  Line 238-252 in statistical analysis part. |
|  | Clarity and Language Quality: The presentation of results is clear, with effective use of tables and figures to summarize findings. The language is mostly clear, though some parts could benefit from more careful proofreading to correct minor grammatical errors and ensure consistency in terminology. | The language in the result part is corrected as advised. | Page 6  Line: 254-262  Page 8:  Line:319-325  Page 9:  355-360 |
|  | Discussion  Relevance: The Discussion makes relevant connections between the findings and broader QoL research. However, it could be improved by including a more comprehensive comparison with existing literature, especially concerning different QoL instruments used in diabetes research. | More comprehensive comparison with existing literature has been done as suggested. | Page 12  line 483-495 |
|  | The Discussion is insightful but occasionally drifts into generalization. More precise language could help in directly linking the study’s results to implications for clinical practice. Enhancements in word choice and sentence structure would also elevate the overall professional quality of the manuscript. | Proof reading done as suggested | Page 12,13  Line: 483-501 |
| Reviewer 2: | Intro: please, add one to two sentences that cover the aim of presented paper. | 1-2 sentences on introduction part added as suggested. | Page 3  Line 120-123 |
|  | Methods: Please, add institutional EC decision # | EC decision # has been kept. | Page 4 Line:168 |
|  | please, explain what the adaptation of questionnaire means? | The word adaptation has been removed. | Page 4 line 177 |
|  | Pearson's correlation coefficient is r. Please, correct. | Has been corrected as suggested. | Page 6 , line:247 |
|  | Results: I mean that in the Tables 1, 3, 4, the column No of respondents (n=151) should be deleted after it is emphasized and explained in the text linked to the tables (i.e. entire study population are the respondents). | The column from table 1,3,4 has been removed and explained in the text linked to tables. | Page 7, 10,11 |
|  | What the letter H means in Mann-Whitney test (it can be U or Z)(the text below the Table 2)? Please, correct or explain if it is possible. | Letter Z was mistakenly written as H.Corrected | Page 9, line 345 |
|  | Add the Pearson's coefficient r in front of -263 in the form: r=-0.263, p<0.00. In presented form, such correlation is not statistically weak, as you mentioned. On contrary, it is a robust negative correlation. Please, correct! | Corrected as suggested | Page 9 , line 350-353 |
